# Notch1 siRNA and AMD3100 Ameliorate Metabolic Dysfunction-Associated Steatotic Liver Disease

**DOI:** 10.3390/biomedicines13020486

**Published:** 2025-02-16

**Authors:** Chunli Zhu, Yiheng Cheng, Lei Yang, Yifu Lyu, Jingjing Li, Pengbo Zhao, Ying Zhu, Xiaofei Xin, Lifang Yin

**Affiliations:** 1 Department of Pharmaceutics, China Pharmaceutical University, Nanjing 210009, China; zhucl@stu.cpu.edu.cn (C.Z.); kevincheng@stu.cpu.edu.cn (Y.C.); yangleisypu@126.com (L.Y.); lyu.yf@stu.cpu.edu.cn (Y.L.); 1520230140@cpu.edu.cn (J.L.); zhaopengbo@stu.cpu.edu.cn (P.Z.); zhuying@stu.cpu.edu.cn (Y.Z.); 2 NMPA Key Laboratory for Research and Evaluation of Pharmaceutical Preparations and Excipients, China Pharmaceutical University, Nanjing 210009, China; 3 Key Laboratory of Drug Quality Control and Pharmacovigilance, China Pharmaceutical University, Nanjing 210009, China; 4 State Key Laboratory of Natural Medicine, China Pharmaceutical University, Nanjing 210009, China

**Keywords:** MASLD, siNotch1 and AMD3100, macrophage phenotype switching, fibrosis and inflammation, cellular crosstalk

## Abstract

**Background and Objectives**: As a key mechanism of metabolic dysfunction-associated steatotic liver disease (MASLD) pathogenesis, inflammation triggered by chronic liver injury and immune cells with macrophages enables MASLD to progress to an advanced stage with irreversible processes such as fibrosis, cell necrosis, and cancer in the liver. The complexity of MASLD, including crosstalk between multiple organs and the liver, makes developing a new drug for MASLD challenging, especially in single-drug therapy. It was reported that upregulation of Notch1 is closely associated with the function of pro-inflammatory macrophages. To leverage this signaling pathway in treating MASLD, we developed a combination therapy. **Materials and Methods**: We chose Notch1 siRNA (siNotch1) to block the Notch pathway so that phenotypic regulation and functional recovery can be achieved in macrophages, combining with small molecule drug AMD3100. AMD3100 can cut off the migration of inflammatory cells to the liver to impede the development of inflammation and inhibit the CXCL12/CXCR4 biological axis in liver fibrosis to protect against the activation of HSCs. Then, we investigated the efficacy of the combination therapy on resolving inflammation and MASLD. **Results**: We demonstrated that in liver cells, siNotch1 combined with AMD3100 not only directly modulated macrophages by downregulating multiple pathways downstream of Notch, exerting anti-inflammatory, anti-migration, and switch of macrophage phenotype, but also modulated macrophage phenotypes through inhibiting NET release. The restored macrophages further regulate HSC and neutrophils. In in vivo pharmacodynamic studies, combination therapy exhibits a superior therapeutical effect over monotherapy in MASLD models. **Conclusions**: These results constitute an siRNA therapeutical approach combined with a small molecule drug against inflammation and liver injury in MASLD, offering a promising therapeutic intervention for MASLD.

## 1. Introduction

Metabolic dysfunction-associated steatotic liver disease (MASLD), renamed from non-alcoholic liver disease (NAFLD), is considered one of the most common causes of chronic liver diseases, including progression of hepatic steatosis, metabolic dysfunction-associated steatohepatitis (MASH), fibrosis, and hepatocellular carcinoma (HCC) [1,2]. Meanwhile, MASLD is a multisystem disease with some extrahepatic complications like cardiovascular disease (CVD), type 2 diabetes mellitus, and chronic kidney disease [3]. MASLD represents a significant and progressively increasing global health and economic burden, while prevalence of MASLD continues to increase substantially worldwide [4]. The good news is that the FDA approved the first drug for the treatment of MASH, Rezdiffra, in 2024, but the long-term efficacy of the drug is still being studied and evaluated [5]. Moreover, other promising drugs targeting mechanisms of MASLD are still under clinical trials. Apart from developing new drugs, considering the complex pathogenesis of MASLD, it is necessary to propose more clinical protocols for drug combination to treat MASLD, as efficacy can be increased and side effects can be reduced in this way [6].

The Notch signaling pathway is extremely evolutionarily conserved and is extensively involved in various diseases of the nervous, immune, and cardiovascular systems [7]. In MASLD progression, the Notch signaling pathway is associated with hepatic lipid accumulation, insulin resistance (IR), oxidative stress (OS), fibrogenesis, and autophagy progression in MASLD [8]. Specifically, the Notch signaling pathway is involved in the activation and effect of pro-inflammatory macrophages, and it directly regulates the transcription of pro-inflammatory signature genes, such as Il6, Il12b, and Nos2 [9]. In monocytes, the Notch signaling pathway plays a crucial role in cell migration and differentiation [10], and it also mediates the transition between the Ly6C^high^ inflammatory phenotype and the Ly6C^low^ circulating surveillance phenotype through mechanisms similar to those in macrophages [11]. Therefore, it is expected that blocking the Notch pathway will stimulate the anti-inflammatory function of macrophages and their differentiation to restorative macrophages. However, gamma-secretase inhibitors targeting the Notch pathway are prone to off-target effects [10]. Compared to small molecules, siRNA can precisely target and regulate target genes due to the mechanism of RNA interference (RNAi) [12]. Therefore, we selected siRNA-Notch1 (siNotch1) to induce specific and effective silence of the Notch1 gene. Currently, AMD3100 is the CXCR4 antagonist, blocking the CXCL12/CXCR4 axis and its signaling pathway [13]. CXCR4 signaling can increase the chemotactic function of inflammatory cells like monocytes and lymphocytes by interacting with CXCL12; thus, homing and migration of inflammatory cells like CD4^+^Tcells to the liver will happen [14]. With inhibition by AMD3100, we can cut off the source of immune cells in the liver to hinder inflammation development. Moreover, CXCL12/CXCR4 also participates in the occurrence of liver fibrosis by promoting the activation and proliferation of hepatic stellate cells [15]. Therefore, blocking the CXCR4 signaling pathway through AMD3100 in hepatic stellate cells may directly inhibit the progression of liver fibrosis. The strategy of targeting the Notch/CXCR4 pathway is expected to play a synergistic role in the direction of inhibiting inflammation-related signaling pathways, macrophage regulation, and fibrosis regulation.

Herein, we present a combination therapeutic approach: co-administration of AMD3100 and siNotch1 for the treatment of MASLD-related liver inflammation, liver injury, and liver fibrosis. Specifically, we applied the combination therapy to achieve regulation on the expression of Notch1 pathway-related genes and protein expression levels in macrophages, switch of macrophage phenotypes from pro-inflammatory to anti-inflammatory, and suppression of macrophage migration. We further investigated the effect of combination therapy on modulating the crosstalk between macrophages and neutrophils, hepatocytes, and hepatic stellate cells (HSCs). Our results demonstrated that this combination therapy not only directly suppressed the activation of HSCs, but can also regulate HSCs and hepatocytes through changing cytokines secreted by macrophages. Interestingly, through inhibiting the release of NETs from neutrophils, combination therapy successfully drove macrophage phenotype-switching. Reversely, the clearance effect of macrophages on NETs can be restored. Using a carbon tetrachloride (CCl_4_)-induced mice model of MASLD, we evaluated the therapeutic effect of combination therapy in vivo and found that compared to monotherapy, co-administration of siNotch1 and AMD3100 exhibited superior therapeutical effects in terms of reducing liver fibrosis and inflammation. Combination of AMD3100 and siNotch1 effectively relieved histopathological changes in the liver. Liver function indicators alanine transaminase (ALT) and aspartate transferase (AST), as well as oxidative stress indicators superoxide dismutase (SOD), catalase (CAT), and glutathione peroxidase (GSH-Px), showed comparable levels as those of healthy mice, representing the recovery of liver cell damage and the enhancement of free radical scavenging ability. This work presents a potent siNotch1 and AMD3100 combination therapy capable of promoting reduction in liver fibrosis and effectively ameliorating fibrosis, lipid accumulation, oxidative stress, and other “multiple-hit” factors of MASLD, offering a promising therapeutic strategy for MASLD patients suffering from liver fibrosis and inflammation.

## 2. Materials and Methods

### 2.1. Materials

Hexadecanoic acid (P651227) was purchased from Aladdin Biochemical Technology Co., Ltd. (Shanghai, China). Negative control siRNA (NC), siRNA Notch1 (siNotch1), and qPCR primers were all obtained from General Biosystems Co., Ltd. (Hefei, China). Taq Pro Universal SYBR qPCR Master Mix (Q712-02) was purchased from Nanjing Vazyme Biotech Co., Ltd. (Nanjing, China). M-CSF (Z03275) and IFN-γ (Z02916) were purchased from Genscript Biotech Co., Ltd. (Nanjing, China). Five units of All-In-One RT master mix (G592) were purchased from Applied Biological Materials Inc. (Vancouver, BC, Canada). A PKH26 red fluorescent cell membrane kit (D0030) and murine bone marrow neutrophil isolation kit (P8550) were purchased from Beijing Solarbio Science & Technology Co., Ltd. (Beijing, China). Phorbol 12-myristate 13-acetate (PMA) (R911744), ferric ammonium citrate (E906531), and oleic acid (D87790) were purchased from Shanghai Macklin Biochemical Technology Co., Ltd. (Shanghai, China). Lipopolysaccharide (LPS) (L2630) was purchased from Sigma-Aldrich Co., Ltd. (St. Louis, MO, USA). Mouse primary antibodies of Notch1 (EP1238Y) (ab52627), Goat Anti-Rabbit IgG H&L (ab6702), and mouse primary antibodies of TGF-β (EPR21143) (ab215715) were purchased from Abcam Trading (Shanghai) Co., Ltd. (Shanghai, China). Mouse primary antibodies of NF-κB (#8242) and α-SMA (#14968) were purchased from Cell Signaling Technology Inc. (Boston, MA, USA). m-IgGκ BP-HRP (sc-516102), mouse antibodies of β-actin (2A3) (sc-517582), and TNF-α (52B83) (sc-52746) were purchased from Santa Cruz Biotechnology Inc. (Dallas, TX, USA). An ECL kit (180-501) was purchased from Tanon Technology Co., Ltd. (Shanghai, China). SuperPAGE Precast Gels (Bis-Tris) (LK310) and Tris/MOPS/SDS Electrophoresis Buffer (20×) (PS120) were purchased from Shanghai Epizyme Biomedical Technology Co., Ltd. (Shanghai, China). RIPA Lysis Buffer (P0013C), DAPI (C1002), protease and phosphatase inhibitor cocktail (P1045), SDS-PAGE loading buffer (P0963), a BCA quantitation kit (P00125), fast blocking buffer (P0020), Western blot enhancer (P0272), polyvinylidene difluoride (PVDF) membrane (FFP22), protein marker (P0060S), and an RNAeasy™ animal RNA extraction kit with a spin column (R0024) were purchased from Beyotime Biotechnology Co., Ltd. (Shanghai, China). Antibodies of FITC anti-Ly6G (BioLegend Cat. No. 127605), PE anti-CD11b (BioLegend Cat. No. 101207), and PE/Cy7 anti-Ly6C (BioLegend Cat. No. 128017) were purchased from BioLegend, Inc. (San Diego, CA, USA). Antibodies of Percp/Cy5.5 anti-F4/80 (Q61594) and EV450 anti-F4/80 (Q61549) were purchased from Elabscience Biotechnology Co., Ltd. (Wuhan, China). SYTOX green nucleic acid stain (KGA1110-20), fetal bovine serum (FBS) (KGL3002-100), and penicillin–streptomycin solution (KGL2303-100) were purchased from Jiangsu Keygen Biotech Co., Ltd. (Nanjing, China). An alanine aminotransferase (ALT/GPT) assay kit (C009-2-1), aspartate aminotransferase (AST/GOT) assay kit (C010-2-1), total superoxide dismutase (SOD) assay kit (A001-3-2), catalase (CAT) assay kit (A007-1-1), and glutathione peroxidase (GSH-Px) (A005-1-2) assay kit were purchased from Nanjing Jiancheng Bioengineering Institute (Nanjing, China).

### 2.2. Cell Culture

AML-12, RAW264.7, and HSC-T6 cells were purchased from Nanjing KeyGEN Biotech Co., Ltd. (Nanjing, China). AML-12 cells were cultured in DMEM/F12 medium supplemented with 10% FBS, 1% (*v*/*v*) penicillin, and 1% (*v*/*v*) streptomycin. RAW264.7 and HSC-T6 cells were cultured in DMEM medium supplemented with 10% FBS, 1% (*v*/*v*) penicillin, and 1% (*v*/*v*) streptomycin. Primary bone marrow cells were cultured in RPMI 1640 medium supplemented with 10% FBS and 1% (*v*/*v*) penicillin and 1% (*v*/*v*) streptomycin. The cells were incubated in a 100% humidified atmosphere with 5% CO_2_ at 37 °C.

### 2.3. Isolation and Differentiation of Primary Mouse Bone Marrow Cells and Neutrophils

Male BALB/c mice with weights of 20–22 g were purchased from Shanghai Sippe-Bk Lab Animal Co., Ltd. (Shanghai, China) and maintained in accordance with “Principles of Animal Experiment Management” and “Guidelines for the Management and Use of Laboratory Animals”. The animals were kept in an environment with a 12-h day/night circle, according to protocols approved by the Institutional Animal Care and Use Committee (IACUC) of the China Pharmaceutical University (Protocol# 2022–05-008 and 2022–05-009).

Firstly, in the biosafety cabinet, we immersed the femur in 75% ethanol, then transferred it to a dish containing cold PBS for washing. Next, both ends of the femur were cut using scissors and a 1 mL syringe was applied to gently flush out the bone marrow cells. The obtained bone marrow cells were filtered through a 70 μm strainer into a 50 mL centrifuge tube and centrifuged at 2500 rpm for 8 min, and then the supernatant was removed. Cell pellets were resuspended in 5 mL sterile red blood cell lysis buffer. After resuspension, cells were incubated at 4 °C for 5 min, diluted with 50 mL of PBS, filtered through a 70 μm cell strainer, and centrifuged at 2500 rpm for 5 min. Finally, the cells were cultured in RPMI 1640 medium supplemented with 10% serum, 1% penicillin–streptomycin, and 10 ng/mL M-CSF until day 6 to obtain bone marrow-derived macrophages (BMDMs). Neutrophils were isolated using the identical procedure with the utilization of a neutrophil isolation kit.

### 2.4. Alternation of Macrophage Phenotypes and Assessment of NET Release

BMDMs were seeded into a 12-well plate at a density of 3 × 10^6^ cells/well and stimulated with LPS and IFN-γ at final concentrations of 100 ng/mL and 50 ng/mL, respectively. The PBS group was used as the negative control. After 24 h of stimulation, six groups were established: PBS-treated BMDMs alone, and PBS, AMD3100, Lipo6000/NC complexes (Lipo6000-NC), Lipo6000/siNotch1 complexes (Lipo6000-siNotch1), and AMD3100+Lipo6000-siNotch1 with LPS and IFN-γ treatment. The drugs were added to the cells according to the set groups for 24 h, in which the final concentration of siNotch1 was 50 nM, and that of AMD3100 was 20 μM. Then, the BMDMs were utilized for flow cytometry analysis, and the corresponding conditioned medium was collected to incubate neutrophils with PKH26 staining and PMA treatment for 3 h in the poly-L-lysine-coated coverslips. The neutrophils were then incubated with 100 μL of SYTOX solution for neutrophil extracellular trap (NET) staining at 37 °C for 15 min. The cells were washed three times with PBS, fixed with 0.5 mL of 4% paraformaldehyde at room temperature for 20 min, and washed again with PBS. DAPI staining solution was added to each sample for 10 min, followed by three washes with PBS. The coverslips were then mounted onto slides with 15 μL of antifade mounting medium and observed under a confocal laser scanning microscope (CLSM).

To evaluate the phenotype of BMDMs under neutrophil-induced inflammation, the BMDMs with LPS and IFN-γ treatment were treated with neutrophil-conditioned medium with PMA induction. Six experimental groups were established: PBS, PBS+LPS+IFN-γ, LPS+IFN-γ+AMD3100, LPS+IFN-γ+Lipo6000-siNotch1, LPS+IFN-γ+AMD3100+Lipo6000-siNotch1. BMDMs were treated with siNotch1 at a final concentration of 50 nM and AMD3100 at 20 μM for 24 h. After incubation, the BMDMs were collected and resuspended in 2% FBS containing antibodies against F4/80, CD11b, and Ly6C at 4 °C for 20 min. After washing the cells with 2% FBS, they were fixed with 4% paraformaldehyde, incubated at room temperature for 30 min, washed again with 2% FBS, and resuspended in 0.5 mL of 2% FBS for flow cytometry analysis.

To further evaluate the capability of NET clearance by BMDMs, neutrophils were firstly resuspended and seeded into a 12-well plate containing poly-L-lysine-coated coverslips at a density of 1 × 10^6^ cells/well. The cells were incubated for 3 h to allow for adherence before being stimulated with PMA at a final concentration of 20 nM for 12 h to induce extensive NET release. Next, BMDMs were seeded into 12-well plates at a density of 3 × 10^6^ cells/well, and then stimulated with LPS and IFN-γ with final concentrations of 100 ng/mL and 50 ng/mL, respectively, for 24 h followed by different treatments, except the PBS group. Five experimental groups were established: PBS, PBS+LPS+IFN-γ, AMD3100+LPS+IFN-γ, Lipo6000-siNotch1+LPS+IFN-γ, and Lipo6000-siNotch1+AMD3100+LPS+IFN-γ. The final concentrations of siNotch1 and AMD3100 were 50 nM and 20 μM, respectively. After 12 h of incubation, BMDMs were gently scraped off and added to the neutrophils containing released NETs for 36 h. Afterwards, the cells were stained with 100 μL of SYTOX staining solution at 37 °C for 15 min, fixed with 0.5 mL of 4% paraformaldehyde at room temperature for 20 min, and washed with PBS. Each sample was then stained with 100 μL of DAPI staining solution for 10 min. The poly-L-lysine-coated coverslips were mounted onto slides with 15 μL of antifade mounting medium and observed using CLSM.

### 2.5. Western Blot and PCR

The RAW264.7 cells were seeded into 6-well plates at a density of 1 × 10^6^ per well with pre-treatment of LPS and IFN-γ for 24 h. We then treated the cells with PBS, AMD3100, Lipo6000, Lipo6000-siNotch1, and Lipo6000-siNotch1+AMD3100 for 24 h, respectively. Proteins were extracted from cells using RIPA buffer which contained protease and phosphatase inhibitors. The total proteins were determined with a BCA protein assay kit, then separated by electrophoresis in SDS-PAGE. Proteins were probed with indicated primary antibodies and secondary antibodies were used to quantify the protein level by the Tanon 4600 SF imaging system (Shanghai, China).

BMDMs were seeded in 6-well plates at a density of 6 × 10^6^ cells/well with LPS and IFN-γ stimulation. The concentration of LPS was 100 ng/mL and that of IFN-γ was 50 ng/mL. Six groups were established: PBS, PBS+LPS+IFN-γ, AMD3100+LPS+IFN-γ, Lipo6000-NC+LPS+IFN-γ, Lipo6000-siNotch1+LPS+IFN-γ, and Lipo6000-siNotch1+AMD3100+LPS+IFN-γ. The drugs were administered to the cells according to the previously established groups, with the final concentration of siNotch1 at 50 nM and the final concentration of AMD3100 at 20 μM, respectively. Following a 24 h incubation period, total RNA extraction, cDNA synthesis, and qPCR were conducted. Total RNA of BMDMs was extracted using the RNAeasy™ extraction kit, then extracted RNA was reverse-transcribed using a HiScript II 1st Strand cDNA Synthesis Kit, and qPCR was performed using Taq Pro Universal SYBR qPCR Master Mix in a Light Cycler 480 (Basel, Switzerland) according to the manufacturers’ instructions. The mRNA levels were normalized with β-actin.

AML-12 cells or HSC-T6 cells were inoculated into a co-culture Transwell system with BMDMs at a density of 5 × 10^5^ cells/mL per well with free fatty acid (FFA) stimulation, respectively. The total final concentration of FFA was 400 μM, in which palmitic acid/oleic acid = 1:2 (mol/mol). BMDMs were treated with PBS, AMD3100, Lipo6000-NC, Lipo6000-siNotch1, and Lipo6000-siNotch1+AMD3100 under LPS and IFN-γ stimulation. After 24 h of administration, total RNA samples were extracted for PCR analysis and total proteins were collected for Western blotting.

### 2.6. Migration Assay

The migration of BMDMs and HSC-T6 were performed following previous studies [16]. Briefly, each Transwell insert was washed twice with PBS and then fixed in 4% paraformaldehyde at room temperature for 30 min. Cells that migrated toward the lower chamber were stained with crystal violet for 10 min. After that, cells were washed by PBS twice and observed using an optical microscope for quantification.

### 2.7. Therapeutic Studies

For the therapeutic study, the mice were randomly divided into 5 groups (n = 6 per group): healthy control, CCl_4_ model, CCl_4_ model treated by AMD3100, CCl_4_ model treated by Liposome-siNotch1, and CCl_4_ model treated by Liposome-siNotch1+AMD3100 through intravenous tail injection every three days a total of five times. siNotch1 was administered at 0.66 OD per mouse and AMD3100 was administered at 3 mg/kg. The treatment was administered once every 3 days for a total of 5 consecutive doses, with the body weight of BALB/c mice recorded throughout the treatment period. After the administration, the heart, liver, spleen, lung, kidney, and blood of the mice were collected for subsequent experiments. The following examination included H&E staining, Sirius red staining, Masson staining, AST/ALT tests, and oxidative stress tests.

### 2.8. Statistical Analysis

Data are presented as means ± standard deviations (SD) by Graphpad prism 10.1.2 except the data in WB. Statistical analysis was performed by one-way analysis of variance. Significant differences are indicated by * *p* < 0.05, ** *p* < 0.01, *** *p* < 0.001, and **** *p* < 0.0001 between groups.

## 3. Results

### 3.1. siNotch1 and AMD3100 Decreased the Expression of Notch Pathway-Related Proteins and Genes in Macrophages

Hepatic macrophages play a key role in regulating MASLD-related immune responses, exerting a bidirectional influence on disease process. They represent the primary targets of siNotch1 and AMD3100 combined regulation. Given the profound impact of the external environment on macrophages, the effects of AMD3100 and siNotch1 on the expression of relevant downstream pathway proteins in RAW264.7 cells were examined by Western blot analysis under LPS+IFN-γ stimulation (Figure S1A). In the PBS group, the Notch pathway was significantly activated, and the expression of the Notch1 protein itself, as well as the downstream inflammatory pathway-related nuclear factor kappa-B (NF-κB), tumor necrosis factor alpha (TNF-α), and transforming growth factor-β (TGF-β), were all significantly upregulated. At this time, free AMD3100 could still reduce the expression of these proteins to a certain extent, but the effect of siNotch was more significant. siNotch not only effectively inhibited the expression of Notch1 protein, but also significantly downregulated the expression of NF-κB, TNF-α, and TGF-β proteins. The combination of siNotch1 and AMD3100 was the most effective. The Western blot results demonstrated that siNotch+AMD3100 was capable of preventing macrophage activation and inhibiting the expression of inflammation-related pathway proteins in inflammatory environments. Furthermore, their combination is a rational approach to regulate macrophages like RAW264.7 cells.

**Figure 1:**
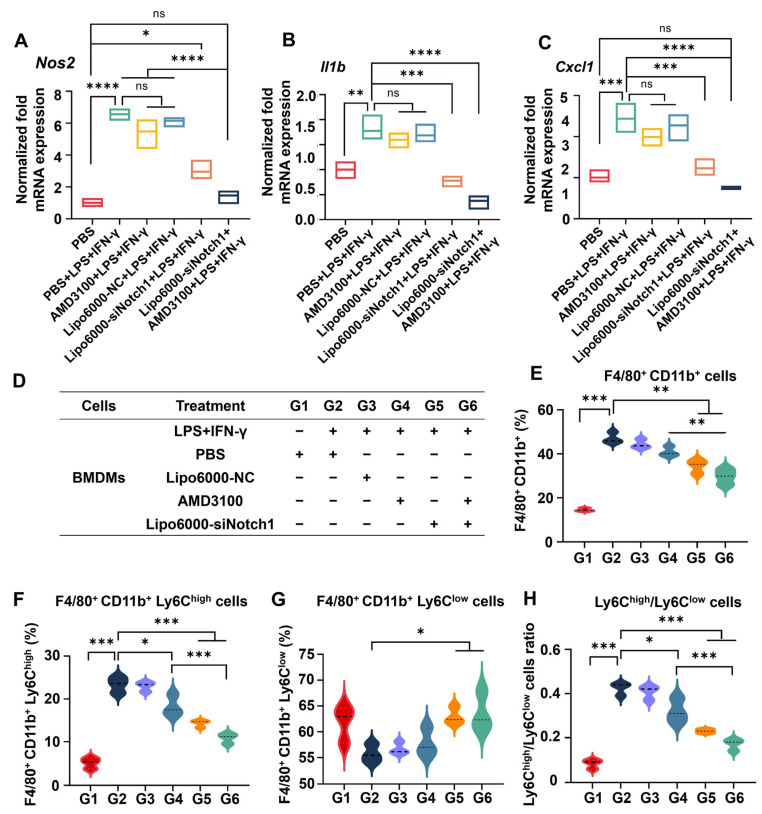
Co-administration of AMD3100 and Lipo6000-siNotch1 inhibits inflammatory activation of BMDMs by blocking the Notch1 signaling pathway and drives macrophage phenotypic switching. (**A**–**C**) The mRNA expression of Nos2, IL-1β, and CXCL1 in stimulated BMDMs treated with AMD3100, Lipo6000-siNotch1, or combination. (**D**–**H**) Quantitative statistics of flow cytometry results of macrophage phenotype switching. The BMDMs were treated with AMD3100, Lipo6000-siNotch1, or combination, stimulated by LPS+IFN-γ. Results are presented as means ± SD (n = 3). ns, not significant. * *p* < 0.05, ** *p* < 0.01, *** *p* < 0.001, and **** *p* < 0.0001, one-way ANOVA test.

Next, we investigated the expression of Notch pathway-related genes by PCR in BMDMs. BMDMs were derived from primary mouse bone marrow cells with the supplement of macrophage colony-stimulating factor (M-CSF) for maturation. Type 2 nitric oxide synthase (Nos2) and interleukin-1beta (IL-1β) were markers of M1-type macrophages [17]. Nos2 has been demonstrated to promote NO synthesis, tumor death, and increasing pathogenic microorganisms, secreting a large number of pro-inflammatory factors, including IL-1β, C-X-C motif chemokine ligand 1 (CXCL1), and others [18,19]. They were expressed in large quantities under the condition of lipopolysaccharide and Interferon-γ (LPS+IFN-γ) stimulation. The results showed that the expression of Nos2, Il1b, and Cxcl1 was significantly downregulated by the combination of siNotch1 and AMD3100 (Figure 1A–C). Furthermore, the frequency of M1-type pro-inflammatory cells was essentially decreased following the administration of AMD3100, which indicated that the inflammatory activation of BMDMs was suppressed and the combination of siNotch1 and AMD3100 had the capacity to influence the phenotype of BMDMs (Figure 1D,E).

Hepatic-infiltrated Ly6C^high^-type macrophages represent pro-inflammatory, pro-tissue damage, and pro-fibrosis roles, while Ly6C^low^-type macrophages play restorative roles in tissue protection, collagen degradation, and fibroblast ablation [20]. To further evaluate the modulation of macrophage phenotype transition by AMD3100 and siNotch1, mouse primary BMDMs were incubated with free AMD310, siNotch1, and their combination, respectively, and Lipo6000 was utilized as the transfection reagent for siNotch1. As shown in Figure 1F–H, the phenotype of BMDMs was significantly affected by siNotch1, which was observed to decrease the ratio of F4/80^+^CD11b^+^Ly6C^high^ cells and increase the ratio of F4/80^+^CD11b^+^Ly6C^low^ cells. Additionally, siNotch1 reduced the Ly6C^high^/Ly6C^low^ ratio. These effects were further ascertained when siNotch1 and AMD3100 were combined. The results demonstrated that siNotch1, in combination with AMD3100, significantly reduced the proportion of pro-inflammatory Ly6C^high^ macrophages and drove macrophage phenotypic switching in BMDMs under the inflammation condition.

### 3.2. siNotch1 and AMD3100 Together Regulated the Crosstalk Between Macrophages and Neutrophils

**Figure 2:**
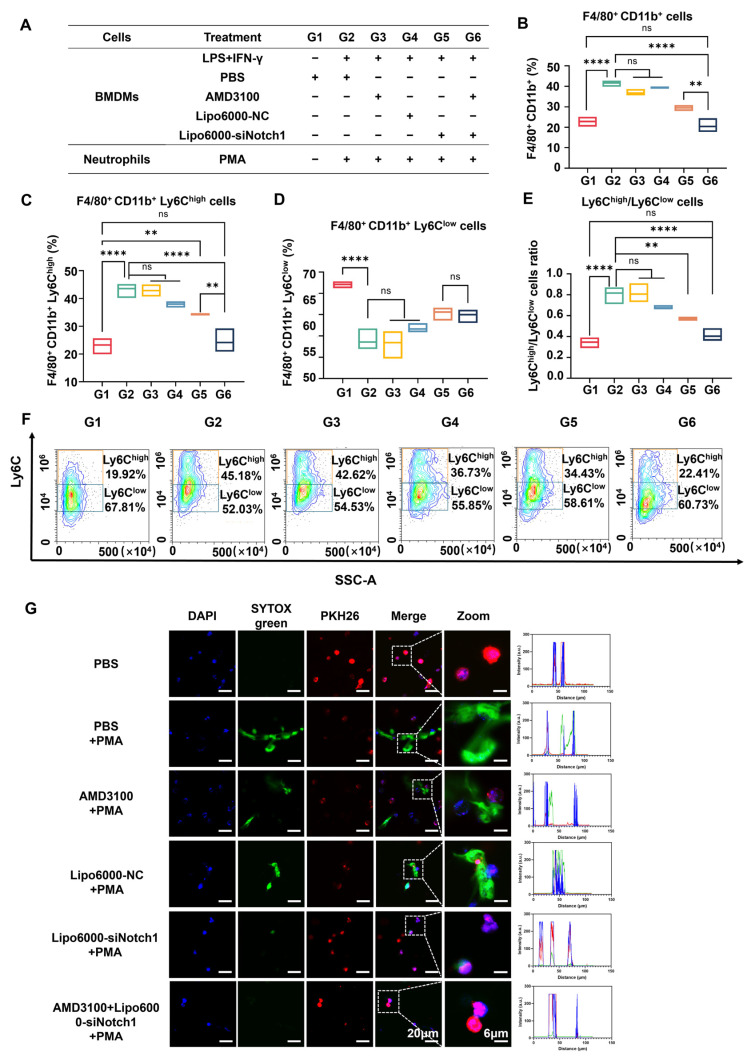
Combined treatment of AMD3100 and Lipo6000-siNotch1 modulates neutrophil–macrophage interactions to mitigate the inflammatory response in MASLD by silencing the Notch1 signaling pathway and blocking the CXCR4 pathway. (**A**–**E**) Quantitative statistics of flow cytometry analysis of F4/80^+^CD11b^+^ cell ratio, F4/80^+^CD11b^+^ Ly6C^high^ cell ratio, F4/80^+^CD11b^+^Ly6C^low^ cell ratio, and Ly6C^high^/Ly6C^low^ cell ratio. (**F**) The proportions of Ly6C^high^-type macrophages and Ly6C^low^-type macrophages in the BMDMs were determined by flow cytometry. (**G**) CLSM images of modulating neutrophil NET release through Lipo6000-siNotch1, AMD3100, or combination, showing NETs (green), neutrophil membrane (red), DAPI (blue), and bright field. Scale bars represent 6 μm and 20 μm. Results are presented as means ± SD (n = 3). ns, not significant. ** *p* < 0.01 and **** *p* < 0.0001, one-way ANOVA test.

Considering that neutrophils have been demonstrated to interact with diverse immune cells like monocytes and macrophages through influencing monocyte differentiation and macrophage polarization, which is significant in the development of MASLD [21], we then evaluated whether the phenotypic switching of macrophages after treatment can modulate the production of neutrophil extracellular traps (NETs), as neutrophils have the ability to promote macrophage differentiation to the Ly6C^low^ type which plays important roles in inhibiting inflammation, promoting wound healing, improving regeneration, and decreasing fiber deposition during tissue injury and fibrosis [22]. Moreover, the uptake of apoptotic neutrophils by macrophages induces the production of anti-inflammatory cytokines (e.g., TGF-β and IL-10) and coordinates anti-inflammatory reprogramming of macrophages [23,24]. Flow cytometry was utilized to investigate the phenotypic regulation of BMDMs by neutrophils under the inflammation condition. The proportion of F4/80^+^CD11b^+^ cells was significantly increased when BMDMs were co-incubated with LPS+IFN-γ stimulation and phorbol 12-myristate 13-acetate (PMA)-induced neutrophil-conditioned medium, indicating that the stimulation conditions were able to promote the macrophage population. The proportion of F4/80^+^CD11b^+^ cells was found to be downregulated following siNotch1 administration, and a further decrease was observed following co-administration with AMD3100 (Figure 2A,B). This suggested that siNotch1 has the potential to regulate macrophage differentiation, engaging in bidirectional interaction with neutrophils. Further study on the effect of different mediums on macrophage phenotype revealed that siNotch1 treatment effectively downregulated the Ly6C^high^ cell frequency and the ratio of Ly6C^high^/Ly6C^low^ cells in BMDMs, and it had a certain synergistic effect with AMD3100 (Figure 2C–E). Figure 2F shows the scatter plots of representative Ly6C^high^ and Ly6C^low^ cell ratios in each group.

Meanwhile, we collected the abovementioned BMDM-conditioned medium with the treatment of siNotch1, AMD3100, and the combination of siNotch1 and AMD3100, to in turn treat PMA-stimulated neutrophils, respectively. The fluorescence of SYTOX Green acted as the marker of neutrophil extracellular traps (NETs), which increase inflammatory cell infiltration and the production of inflammatory cytokine in MASLD progression [25]. Compared with the PBS group, PMA-treated neutrophils released NETs in large quantities. AMD3100 treatment alone failed to inhibit the release of NETs. This result suggested that blockade of the CXCR4 pathway was not sufficient to resist PMA-induced damage to neutrophils. However, siNotch1 decreased NET formation following the stimulation of neutrophils with PMA. The combination of AMD3100 and siNotch1 was confirmed to further inhibit the release of NETs (Figure 2G), indicating that the silence of Notch signaling is necessary for initiation of the CXCR4 blockade in NET release.

Of note, one of the main ways to clear NETs is the phagocytic function of macrophages [26]. Hepatic macrophages in patients with MASLD can be activated and experience functional alterations, accompanied by a reduced ability to clear NETs [27]. To investigate whether siNotch1 and AMD3100 can restore the ability of macrophages to remove NETs, BMDMs with different treatments were co-cultured with NETs released by neutrophils. The results showed that all treatment group restored BMDMs’ clearance effect on NETs. Specifically, the siNotch1 and AMD3100 combination group restored BMDMs’ ability to clear NETs to the normal level (Figure S2). This bidirectional modulation holds promise. These results validated the hypothesis that siNotch1 and AMD3100 can alter the crosstalk between neutrophils and macrophages, thereby providing a foundation for promoting mutual interactions toward inflammation alleviation in MASLD.

### 3.3. siNotch1 and AMD3100 Suppressed the Migration of BMDMs and Restored the Hepatocyte Function

**Figure 3:**
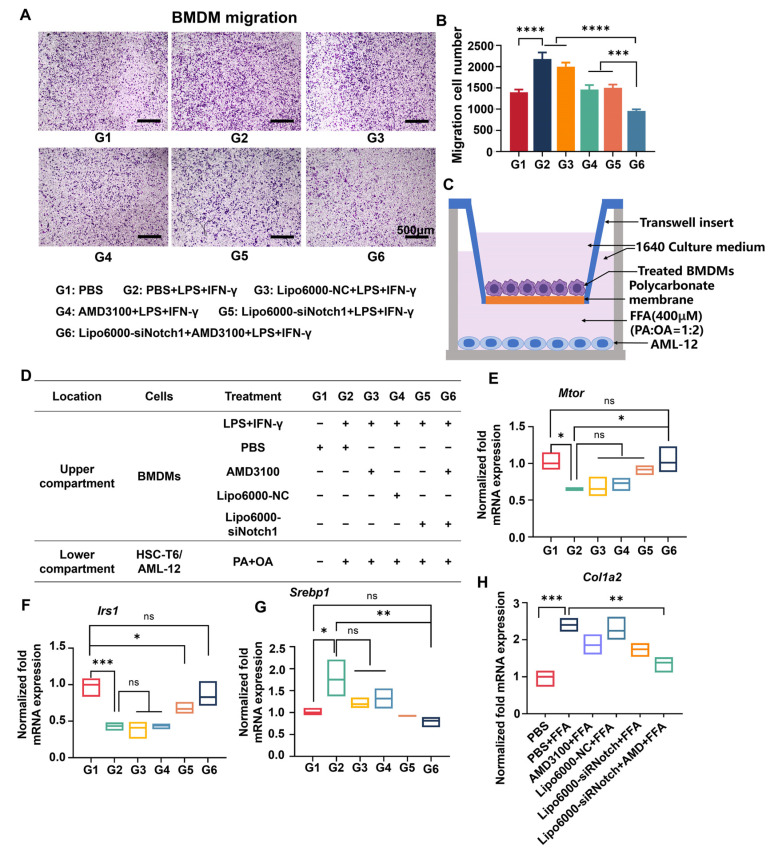
Co-administration of AMD3100 and Lipo6000-siNotch1 inhibits macrophage migration and regulates the cytokine secretion of macrophages to modulate gene expression in hepatocytes, alleviating the progression of MASLD and liver fibrosis. (**A**) Microscopy images of stained BMDMs that invaded through the Matrigel mix and migrated from the top chamber to the bottom chamber in response to LPS+IFN-γ stimulation following indicated treatments. (**B**) Quantitative statistics of BMDMs in the Transwell assay. (**C**) Schematic illustration of the Transwell co-culture system investigating the impact of macrophage-secreted factors in AML-12 cells, using a two-compartment Boyden chamber system. BMDMs were incubated with LPS+IFN-γ, PBS, AMD3100, Lipo6000-NC, and Lipo6000-siNotch1 in the top chamber, and the Culture medium in the bottom chamber was supplemented with chemotactic stimuli PA+OA. Cytokines secreted by BMDMs invaded through themembrane into the bottom chamber. (**D**–**H**) mRNA expression of *Mtor*, *Irs1*, *Srebp1* and *Col1a2*, in stimulated AML-12 cells treated by cytokines from BMDMs receiving indicated treatments. Results are presented as means ± SD (n = 3). ns, not significant. * *p* < 0.05, ** *p* < 0.01, *** *p* < 0.001, and **** *p* < 0.0001, one-way ANOVA test.

During the development of MASLD, hepatic macrophages are recruited by chemokines and migrate to sites of inflammation. The formation of inflammatory foci will exacerbate local hepatocellular injury and promote fibrogenesis, contributing to the aggravation of MASLD [28]. Here, we examined the effect of AMD3100 and siNotch1 on the migration of proinflammatory BMDMs via a Transwell assay. Figure 3A,B show that under LPS+IFN-γ stimulation, the migratory rate of BMDMs was increased and the number of crystal violet-stained cells was significantly increased, indicating that the activated macrophages had stronger migration ability. Intriguingly, free AMD3100 and siNotch1 treatments significantly decreased the migratory rate of LPS+IFN-γ-stimulated BMDMs, respectively. Moreover, co-administration of AMD3100 and siNotch1 exerted the best anti-migration effect. The results revealed that effective suppression of the CXCL12/CXCR4 axis and Notch pathway through AMD3100-assisted siRNA therapy is promising for the inhibition of macrophage migration to inflammatory sites, thereby preventing hepatic fibrosis and alleviating MASLD.

To verify that, we investigated the impact of macrophage-secreted factors on gene expression in AML-12 cells using a Transwell system (Figure 3C) and simulated “fatty” conditions by stimulating AML-12 cells in the lower chamber under the induction of palmitic acid (PA) and oleic acid (OA). Macrophages modulate hepatocytes by secreting various cytokines such as TGF-β, IL-1β, and TNF-α [29]. BMDMs from the upper chamber were treated with different drugs, and the secreted factors diffused through the Transwell membrane to the lower chamber. Figure 3D–G show the gene expression related to the insulin pathway, including *Mtor*, *Irs1*, and *Srebp1* in AML-12 cells. The restoration of *Mtor* and *Irs1* expression promoted normal energy metabolism pathways and restored insulin sensitivity in parenchymal cells. Downregulation of *Srebp1* is beneficial in reducing cholesterol accumulation in the liver, alleviating excessive fat deposition [30]. Next, the mRNA expression of collagen type I alpha 2 chain (Col1a2) in AML-12 cells was examined (Figure 3H). *Col1a2* is pro-alpha2 chain of type I collagen, a fibril-forming collagen found in most connective tissues, which is the primary constituent of ECM in hepatic fibrosis [31]. AMD3100 moderately downregulated *Col1a2* mRNA expression. However, the most significant downregulation was observed in the combination treatment group, further confirming the synergistic effect of AMD3100 and siNotch1 in regulating hepatic fibrosis in AML-12 cells. The observed gene expression changes in AML-12 cells highlight the intricate interplay between BMDMs and hepatocytes, supporting the rational and effective regulation achieved by combination of AMD3100 and siNotch1 acting on macrophages for MASLD therapy.

### 3.4. siNotch1 and AMD3100 Modulated Macrophage–HSC Crosstalk

**Figure 4:**
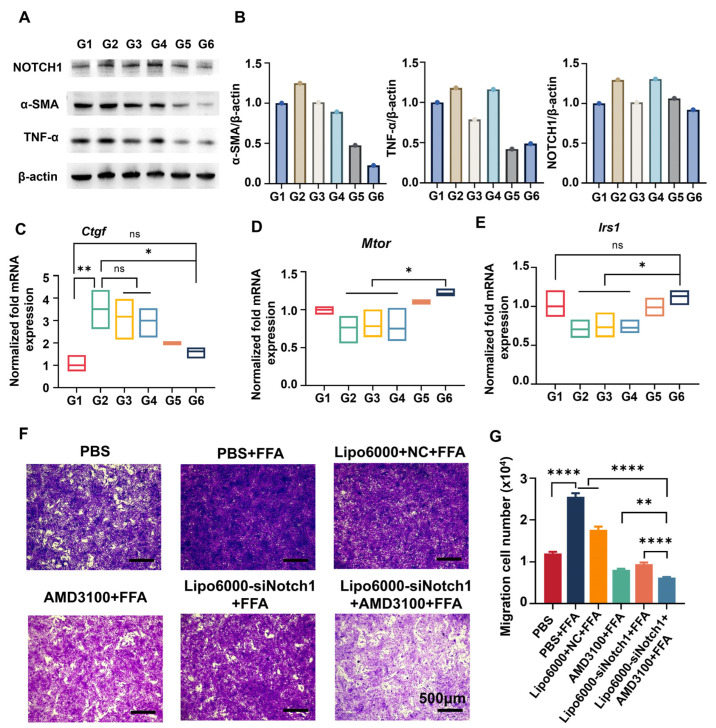
Combined treatment of AMD3100 and Lipo6000-siNotch1 inhibits macrophage-mediated migration and activation of HSCs. (**A**) Western blots of Notch1, α-SMA, and TNF-α in FFA-stimulated HSC-T6 cells co-cultured with BMDMs receiving treatment of AMD3100, Lipo6000-siNotch1, or combined. (**B**) Quantitative analysis of protein levels based on the grey value of the blots in (**A**). (**C**–**E**) mRNA expression of mTOR, IRS1, and Ctgf in the HSC-T6 cells. Results are presented as means ± SD (n = 3). ns, not significant. * *p* < 0.05 and ** *p* < 0.01, one-way ANOVA test. (**F**) Microscopy images of stained HSC-T6 cells that invaded through the Matrigel mix and migrated from the top chamber to the bottom chamber in response to FFA stimulation following indicated treatment. (**G**) Quantitative statistics of HSC-T6 cells in Transwell assay. Results are presented as means ± SD (n = 3). ** *p* < 0.01 and **** *p* < 0.0001, one-way ANOVA test.

Activated hepatic stellate cells (HSCs) are the primary source of the extracellular matrix (ECM), directly leading to excessive ECM deposition and promoting fibrosis progression. Likewise, we studied the expression levels of relevant proteins and genes in HSC-T6 cells when co-cultured with BMDMs with the treatment of AMD3100 and siNotch1, using the Transwell model in Figure 3C. As shown in Figure 4A,B, under fatty acid stimulation, increased protein expression levels of Notch1, α-SMA, and TNF-α were associated with hepatic stellate cell (HSC) activation. AMD3100 alone had a suppressive effect on the expression of α-SMA and TNF-α proteins, consistent with its mechanism of inhibiting HSC activation by blocking the CXCL12/CXCR4 axis. When AMD3100 was used in combination with siNotch1, the expression levels of these proteins significantly decreased, indicating a strong synergistic effect in inhibiting HSC activation.

Connective tissue growth factor (CTGF) is a crucial fibrogenic protein produced by HSCs, and its sustained expression is considered a key factor in fibrosis [32]. Upon combined treatment of siNotch1 and AMD3100 in upper-chamber BMDMs, the expression of Ctgf significantly decreased in lower-chamber HSC-T6 cells (Figure 4C). In the MASLD environment, various pathways related to glucose and lipid metabolism in these cells become abnormal, severely impairing energy metabolism and leading to systemic metabolic dysfunction. Blocking the Notch and CXCR4/CXCL12 pathways in macrophages inhibited the expression levels of mTOR and IRS1, which are key components of the insulin signaling pathway in HSCs (Figure 4D,E).

On the other hand, during the transition from a quiescent to an activated state, HSCs undergo stages of inflammation, migration, and ECM production. Hence, inhibiting the migration of HSCs is a crucial step in hindering the progression of MASLD [33]. Therefore, we collected HSC-T6 cells from the lower chamber to assess the migration capacity of HSCs. Figure 4F,G show that BMDMs with AMD3100 or siNotch1 administrated alone exhibited anti-migratory effects on HSCs. When used in combination, this effect becomes more pronounced, also indicating a strong synergistic interaction between AMD3100 and siNotch1 in macrophage-mediated HSC modulation.

### 3.5. Combination of AMD3100 and siNotch1 Ameliorates Inflammation, Fibrosis, and Oxidative Stress in a CCl_4_-Induced MASLD Model

**Figure 5:**
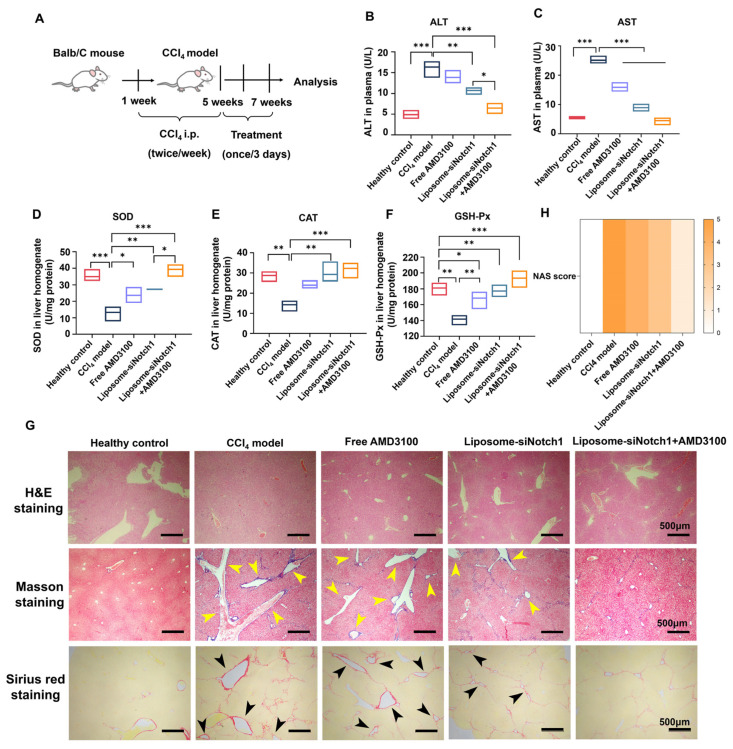
Combination of AMD3100 and Lipo6000-siNotch1 shows superior in vivo therapeutical effect on hepatic inflammation, fibrosis, and oxidative stress in a CCl_4_-induced MASLD model. (**A**) Schematic diagram of the CCl_4_-induced MASLD model and treatment timeline for saline, AMD3100, Lipo6000-siNotch1, or combined therapy. (**B**,**C**) ALT and AST levels in the serum following indicated treatments. (**D**–**F**) SOD, CAT, and GSH-Px levels in the liver homogenate following indicated treatments. (**G**) H&E staining, Masson staining, Sirius red staining, and (**H**) NAS score of liver tissue section, using the NAFLD Activity Score (NAS) proposed by the American Association for the Study of Liver Diseases (AASLD) in 2005 as the pathological diagnostic scoring criterion for MASLD. Results are presented as means ± SD (n = 6). * *p* < 0.05, ** *p* < 0.01, and *** *p* < 0.001, one-way ANOVA test.

We first investigated pathological changes in liver tissue through H&E staining, such as cellular damage, lipid accumulation, and fibrous deposition in the case of MASLD. After being induced by CCl_4_, mouse liver tissue exhibited various pathological changes such as cellular damage, lipid accumulation, and fibrotic deposition, leading to significant alterations in tissue structure and cellular morphology, which mirrored the pathogenesis of human MASLD and thus was used as a clinically relevant model for in vivo efficacy evaluation [34]. CCl_4_-induced mice were characterized with higher ALT and AST levels in liver homogenate. AST levels were elevated 4.5-fold relative to the healthy group, significantly higher than ALT (3.3-fold), and were ameliorated in animals receiving AMD3100 or siNotch1 therapeutics (Figure 5B,C). Among the treatment groups, the downregulation of AST was more pronounced compared to ALT, suggesting that mild liver cell injury persisted following treatment because ALT is a specific marker of liver cell damage [35], but serious mitochondrial damage had been alleviated since increasing the level of AST mainly results from mitochondrial dysfunction in MASLD [36]. Moreover, the level of AST/ALT decreasing from 1.5 to 0.7 illustrated that the liver recovered from advanced fibrosis/cirrhosis after treatment by siNotch1 and AMD3100 [37]. Particularly, in the AMD3100 and siNotch1 combined treatment group, plasma AST and ALT levels had largely returned to the normal level (Figure 5B,C). We also analyzed superoxide dismutase (SOD), catalase (CAT), and glutathione peroxidase (GSH-Px) levels in liver homogenates to evaluate the effects of AMD3100 and siNotch1 on oxidative stress in the CCl_4_-induced mouse model. The occurrence of oxidative stress can further exacerbate damage to various liver cells, leading to cellular apoptosis and necrosis, which promote the recruitment and aggregation of inflammatory cells, thereby amplifying inflammation in MASLD [2]. As shown in Figure 5D–F, AMD3100 and siNotch1 combined therapy successfully reversed the downregulation of SOD, CAT, and GSH-Px to normal levels. These results revealed that co-administration of AMD 3100 and siNotch1 can reverse progression of hepatic inflammation and fibrosis, and inhibit oxidative stress in a murine model induced by CCl_4_, thus elucidating why AMD3100 and siNotch1 combined therapy is more effective.

Histopathological analysis of liver sections showed morphological changes and structural alteration along with swelling, loose arrangement, and unclear cell boundaries in MASLD mice receiving CCl_4_ treatment compared with the healthy control (Figure 5G). After administration of AMD3100 and siNotch1, the morphology of liver tissue cells showed varied degrees of recovery. Notably, in the group treated with AMD3100 and siNotch1 combined, the liver cells exhibited good morphology, with fewer vacuoles and reduced swelling, as well as decreased inflammatory cell infiltration (Figure 5G). Next, Masson’s trichrome and Sirius red staining revealed that the fibrotic regions were markedly increased in the livers of MASLD mice treated with CCl_4_ compared with healthy mice. CCl_4_ induced severe collagen fiber deposition in the liver of model mice, with the most significant fibrosis occurring in the portal area and noticeable bridging fibrosis between liver lobules. Treating the CCl_4_-induced mice with siNotch1 or AMD3100 alone decreased the aniline blue- or Sirius red-positive area in liver tissues. Specifically, collagen fiber deposition around the blood vessels in MASLD mice receiving AMD3100 and siNotch1 co-administration was significantly relieved, although bridging fibers were still presented (Figure 5G and Figure S3). This indicated that in the CCl_4_-induced MASLD model, collagen fiber deposition was difficult to completely eliminate, and the administration of AMD3100 and siNotch1 can reverse this condition. The NAS score results in Figure 5H indicate that the combination of siNotch1 and AMD3100 exhibited the optimal therapeutic effect, significantly improving the pathological state of liver tissue by addressing hepatocyte ballooning, inflammation, and steatosis. This suggested that co-administration of AMD3100 and siNotch1 can effectively alleviate pathological changes in liver tissue cells. The reversing trend of liver damage biomarkers was consistent with liver organ coefficients and histopathological findings, further confirming the superior therapeutic efficacy of AMD3100 and siNotch1 co-administrated.

## 4. Discussion

The global prevalence of MASLD has risen sharply in recent decades, and it has now become the most common chronic liver disease worldwide [38]. The pathogenesis of MASLD is highly complex, and its exact mechanisms remain unclear. As our understanding of the disease deepens, the “multiple-hit” hypothesis, which evolved from the “two-hit” hypothesis, has gradually gained attention [39,40,41]. Despite MASLD threatening the health of nearly one-third of the global population, only one drug has been approved by the U.S. Food and Drug Administration (FDA) for its treatment. The complex pathogenesis poses a significant challenge for the clinical development of drugs for MASLD, leading to the termination of numerous clinical trials, primarily due to insufficient efficacy and an inability to meet the pre-designed clinical endpoints, especially in improving liver histological markers such as fibrosis, which remains a major reason for the failure of MASLD-related drug trials, like Selonsertib (GS-4997) [42] and Elafibranor [43]. The insufficient efficacy of numerous monotherapies in clinical trials not only suggests that our understanding of MASLD’s pathogenesis is still inadequate but also highlights that targeting a single pathway is insufficient to reverse the disease’s progression. The synergistic effect of combination therapies holds promise for achieving better outcomes in the treatment of MASLD. Our study suggests that combined treatment by siNotch1 and AMD3100 can improve “multiple-hit” factors of MASLD, especially in liver inflammation and liver fibrosis. Accordingly, the results emphasize the possibility of AMD3100 and siNotch1 as a therapeutic intervention for MASLD.

Previous studies have demonstrated that chemokine receptor CXCR4 and its ligand in liver disease are very important, playing a key role in multiple liver diseases such as hepatitis, liver injury and regeneration, liver fibrosis, and cirrhosis, as well as in HCC [14]. Boujedidi et al. demonstrated that as a CXCR4 antagonist, AMD3100 inhibits the chemotaxis of CXCL12 to CD4^+^ T cells and reduces the number of CD4^+^ T cells entering the liver [44]. Liu et al. found that AMD3100, as a targeting moiety, suppresses the progression of fibrosis by inhibiting the proliferation and activation of HSCs [45]. There are also some studies reporting that inhibition of the Notch signaling pathway may be effective in alleviating MASLD through reducing secretion of inflammatory cytokines, inhibiting fibroblast activation and promoting macrophage phenotypic switch to an anti-inflammatory phenotype [46,47]. This evidence supports that the inhibition of CXCR4 and the Notch signaling pathway may be a promising strategy for treating MASLD. Many studies have investigated the Notch/CXCR4 partnership in different diseases [48,49] based on the fact that the two signaling pathways interact with each other.

The essential result of the present study is that inhibiting the Notch signaling pathway and the CXCR4/CXCL12 axis are central to MASLD progression, improving macrophage polarization, inflammation, and fibrosis. siNotch1 effectively silenced the Notch1 gene, reducing the expression of pro-inflammatory macrophage markers like Nos2 and Il1b (Figure 1A–C) and downstream inflammatory markers such as NF-κB, TNF-α, and TGF-β (Figure S1). Meanwhile, AMD3100, a CXCR4 antagonist, further enhanced the effect of combined therapy in inhibition on BMDM migration (Figure 3A) and HSC activation (Figure 4A,B). The combination of these two agents led to a more pronounced reduction in the pro-inflammatory macrophage phenotype and an increase in the anti-inflammatory macrophage population (Figure 2).

Notably, this dual-target approach facilitated crosstalk modulation between macrophages and other cell types. We found that the inhibition of NET release through combination therapy can reduce the generation of pro-inflammatory macrophages, ultimately decreasing inflammation and macrophage chemotaxis (Figure 2A–F). Previous studies have demonstrated that neutrophils are crucial in macrophage polarization. By releasing the neutrophil granule proteins azurocidin, HNP1-3, and NETs, macrophages can be induced to a pro-inflammatory phenotype [50,51], which are characterized by secreting pro-inflammatory cytokines, thus aggravating MASLD progression. Meanwhile, it has also been proved that viable neutrophils can facilitate macrophage switching to anti-inflammatory phenotype and reduce pro-inflammatory cytokine release [52]. As for the effect of polarized macrophage in NET degradation, previous studies have illustrated that NETs can be degraded by the cytosolic exonuclease TREX1 in macrophages [53]. Furthermore, continuous pro-inflammatory polarization leads to dramatically reduced stimulated macropinocytosis in macrophages [54]. In this study, we successfully reversed the continuous polarization of macrophages under the inflammatory condition through the switch of macrophage phenotype to anti-inflammatory, thus facilitating clearance of NETs by macrophages (Figure S2). Ultimately, we achieved bidirectional regulation between macrophages and neutrophils, thereby exerting an inhibitory effect on inflammation. However, it is also reported that when anti-inflammatory macrophages interact with NETs, they induce several pro-inflammatory cytokines/chemokines, leading to an inflammatory response that, in turn, enhances the infiltration of other inflammatory cells [55]. The impact of this factor on therapeutic effects requires further investigation in future studies. As key regulators in MASLD, we also observed extensive interactions between macrophages and other hepatic cells. Previous research found that the pro-inflammatory macrophage population was positively correlated with insulin resistance and increases through targeting FFA-mediated pro-inflammatory responses, dominating under conditions of nutrient excess [56]. We inferred that keeping the balance of polarized populations of macrophages can be helpful for normalization of insulin sensitivity in MASLD. Our research has shown that factors secreted by macrophages after treatment of combined therapy can improve the energy metabolism mode in hepatocytes where the expression of genes associated with insulin resistance has returned to normal, then promote insulin sensitivity, thereby alleviating fat accumulation in the liver (Figure 3D–G). CTGF is an important fibrosis-related protein [32] and was significantly reduced under the combined treatment, indicating that this therapeutic strategy effectively slows down fibrosis progression. Furthermore, the study also found that combination therapy inhibited the migration of HSCs (Figure 4F,G) and restored the expression level of insulin resistance-related genes to normal (Figure 4C–E). Lastly, we evaluated the in vivo therapeutic effects of combined therapy in a well-established murine model of MASLD induced by CCl_4_. Compared with the MASLD model without treatment, AMD3100 and siNotch1 reversed liver cell damage, oxidative stress, and mitochondrial dysfunction according to the ameliorating levels of ALT, AST, SOD, CAT, and GSH-PX (Figure 5B–F). Meanwhile, it can be calculated from the histopathological analysis that inflammatory cell infiltration and liver fibrosis significantly improved (Figure 5F,G and Figure S3).

In summary, since MASLD is a multifactorial disease involving multiple cell signaling pathways and organs, co-administration of two drugs will have a significant impact on the treatment of MASLD. Especially in the current paucity of new drugs for MASLD, the development of a co-administration strategy using previously developed drugs targeting relevant pathways may be effective in improving the limited therapeutic options for MASLD. However, the choice of delivery systems for different drugs still requires careful consideration in clinical practice. Furthermore, recent studies have discovered that the mammalian circadian clock is closely associated with liver physiology and disease by controlling hepatic uptake transporters, hepatic metabolism, and transcription factors, especially in fatty liver diseases like MASLD [57]. Therefore, we may need to adjust the timing of the combined administration based on the biological clock to ensure the maximum therapeutic benefit and safety of the medication. For instance, macrophages have a circadian clock that regulates the rhythmic secretion of TNF and IL-6 [58], which leads to macrophages exhibiting a marked circadian rhythmic pattern in the secretion of TNF-α and IL-6, with a peak-to-trough ratio of approximately 3 [59]. At the same time, it has been reported that perturbation of circadian rhythmicity in HSCs correlates with fibrotic gene expression in vivo and activation of the circadian clock component REV-ERB can suppress TGF-β signaling in liver disease and may attenuate the progression of fibrosis [60]. These studies on the circadian clock provide us with inspiration, suggesting that in the future, we may develop combination therapies that include circadian clock-targeting modulators to treat MASLD through a multi-target approach.

## 5. Conclusions

Overall, our findings present a robust combination therapy centered on regulating liver inflammation to treat MASLD through co-administration of siNotch1 and AMD3100 simultaneously suppressing the Notch1 signaling pathway and CXCR4. In terms of mechanism, combination therapy can regulate macrophage phenotype and function in vitro through modulating multi-level interactions between macrophage and other cells, thereby facilitating inflammation resolution and promoting alleviation of fibrosis and insulin resistance. Furthermore, we investigated its therapeutical effect in a CCl_4_-induced murine model of MASLD to prove that combination therapy is capable of improving “multiple-hit” factors of MASLD. This work demonstrates the promising translational potential of siRNA-based therapy for MASLD and other liver diseases including related hepatic fibrosis.

## Data Availability

Dataset available on request from the authors.

## Supplementary Materials

The following supporting information can be downloaded at: https://www.mdpi.com/article/10.3390/biomedicines13020486/s1, Figure S1: Combined treatment of AMD3100 and Lipo6000-siNotch1 suppresses inflammatory activation in macrophages through inhibiting Notch1 pathway-related proteins; Figure S2: Combination of Lipo6000-siNotch1 and AMD3100 restores the ability of BMDMs to clear NETs to normal levels; Figure S3: Fibrosis stage of liver tissue section, using the SAF (Steatosis, Activity and Fibrosis) Score proposed by the European Association for the Study of the Liver (EASL) in 2012 as the scoring criteria of fibrosis stage for MASLD; Table S1: Primer sequence.
