# Supplementary material for: Notch1 siRNA and AMD3100 Ameliorate Metabolic Dysfunction-Associated Steatotic Liver Disease
Source: Biomedicines. 2025 Feb 16;13(2):486. doi: 10.3390/biomedicines13020486 (PMC11853639; doi:10.3390/biomedicines13020486)
Supplement: Supplementary file 1 [file biomedicines-13-00486-s001.zip › biomedicines-3411646-supplementary.pdf]

Supplementary Materials

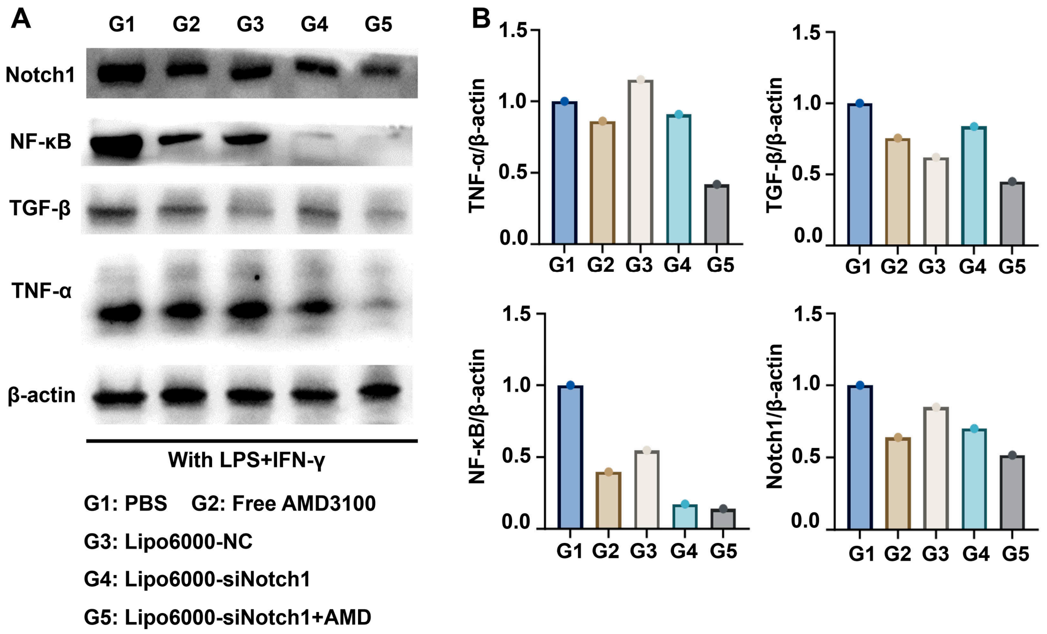

Figure S1. Combined treatment of AMD3100 and Lipo6000-siNotch1 suppresses inflammatory activation in macrophages through inhibiting Notch1 pathway-related proteins. (A) Western-blot results of Notch1, NF-κB , TNF-α and TGF-β proteins in stimulated BMDMs treated with AMD3100, Lipo6000-siNotch1 or combination. (B) Relative quantitative analysis of the expression levels of Notch1, NF-κB, TNF-α and TGF-β proteins in macrophages across different groups.

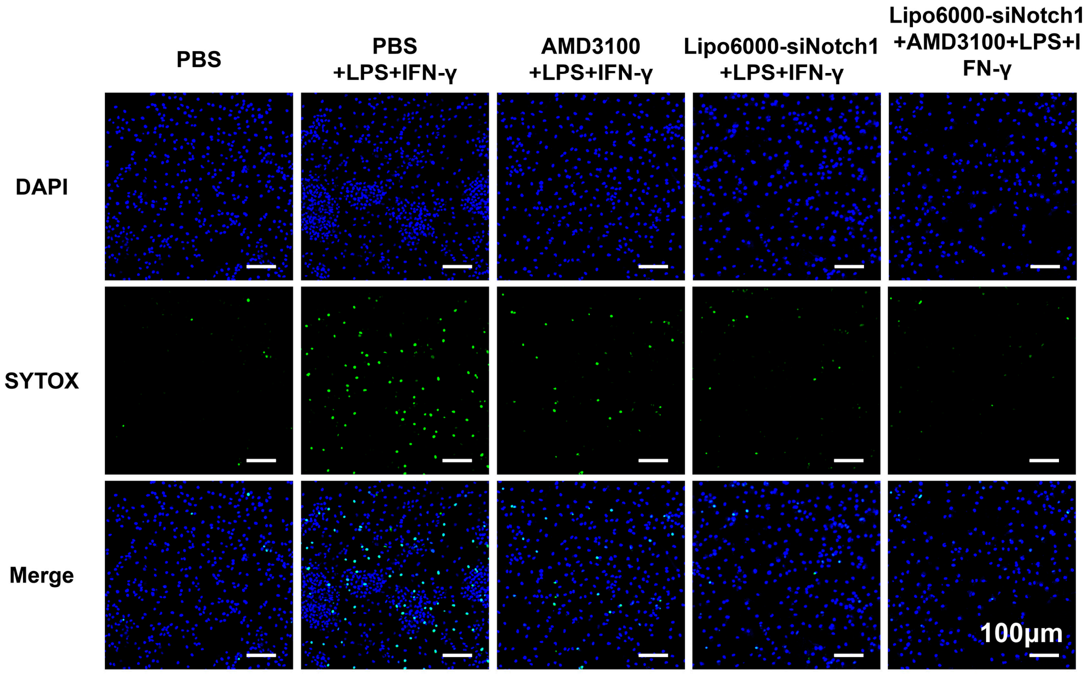

Figure S2. Combination of Lipo6000-siNotch1 and AMD3100 restores the ability of BMDMs to clear NETs to normal levels. The figure presents CLSM images of the NETs released by PMA induced neutrophils co-incubated with stimulatd BMDMs treated with AMD, Lipo6000-siNotch1 or both, showing DAPI (blue) and SYTOX (green), scale bar represents 100μm.

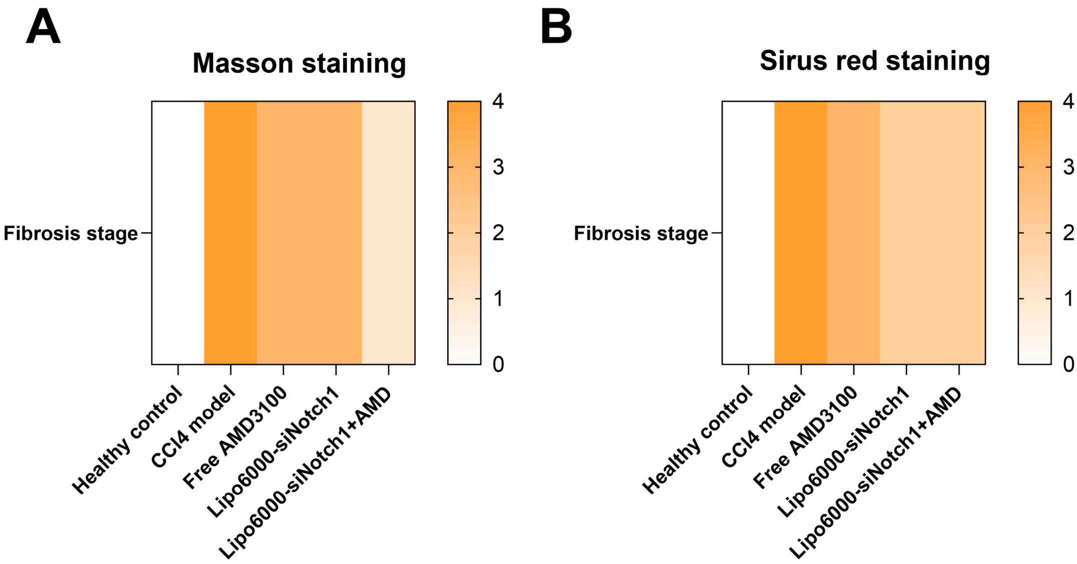

Figure S3. Fibrosis stage of liver tissue section, using the SAF (Steatosis, Activity and Fibrosis) Score proposed by the European Association for the Study of the Liver (EASL) in 2012 as the scoring criteria of fibrosis stage for MASLD. n=6 mice per group.

Table S1. Primer sequence

Gene	Primer	Sequence (5'-3')
Rat *Colla2*	Forward	ACCTCAGGGTGTTCAAGGTG
	Reverse	CGGATTCCAATAGGACCAGA
Rat *Ctgf*	Forward	GCTGACCTAGAGGAAAACATTAAGA
	Reverse	CCGGTAGGTCTTCACACTGG
Rat *β-actin*	Forward	CCGTAAAGACCTCTATGCCA
	Reverse	AAGAAAGGGTGTAAAACGCA
	Forward	ACCGGCACACATTTGAAGAAG
Mouse *Mtor*	Reverse	CTCGTTGAGGATCAGCAAGG
	Forward	CGATGGCTTCTCAGACGTG
Mouse *Irsl*	Reverse	CAGCCCGCTTGTTGATGTTG
	Forward	GCAACTGTTOCTGAACTCAACT
Mouse *Il1b*	Reverse	ATCTTTTGGGGTCCGTCAACT
	Forward	CTGGGATTCACCTCAAGAACATC
Mouse *Cxcl1*	Reverse	CAGGGTCAAGGCAAGCCTC
	Forward	GCAGCCACCATCTAGCCTG
Mouse *Srebp1*	Reverse	CAGCAGTGAGTCTGCCTTGAT
	Forward	GTTCTCAGCCCAACAATACAAGA
Mouse *Nos2*	Reverse	GTGGACGGGTCGATGTCAC
	Forward	CATGTACGTTGCTATCCAGGC
Mouse *β-actin*	Reverse	CTCCTTAATGTCACGCACGAT
